# CGRP remodels the acidic microenvironment and promotes pancreatic carcinoma progression by regulating NHE1 and CA9

**DOI:** 10.3389/fphar.2026.1928812

**Published:** 2026-10-01

**Authors:** Sa Liu, Shanshan Wei, Taoli Sun, Dai Li, Yuanjian Li, Bikui Zhang, Yangchun Xie, Wenqun Li

**Affiliations:** 1 Department of Pharmacy, The Second Xiangya Hospital, Central South University, Changsha, China; 2 Institute of Clinical Pharmacy, Central South University, Changsha, China; 3 Department of Pharmacology, School of Pharmacy, Guangxi Medical University, Nanning, China; 4 Institute of Dietotherapy and Herbal Medicinal Diet, Hunan Academy of Chinese Medicine, Changsha, China; 5 Phase I Clinical Research Center, Xiangya Hospital, Central South University, Changsha, China; 6 Department of Pharmacology, School of Pharmaceutical Science, Central South University, Changsha, China; 7 Department of Oncology, The Second Xiangya Hospital, Central South University, Changsha, China

**Keywords:** acidic microenvironment, CA9, CGRP, NHE1, pancreatic ductal adenocarcinoma, rimegepant

## Abstract

**Introduction:**

Neurons can regulate cancer cells to modulate their survival and growth, the interplay between calcitonin gene-related peptide (CGRP) and the acidic tumor microenvironment (TME) in pancreatic ductal adenocarcinoma (PDAC) remains unknown.

**Methods and Results:**

Here, we reported that CGRP expression and neuronal activation markers p-ERK/p-CREB were significantly elevated in PDAC tissues. Exogenous CGRP promoted PANC02 cell proliferation and migration, whereas CGRP knockout suppressed orthotopic tumor growth. Mechanistically, CGRP upregulated the proton transporters NHE1 and CA9, facilitating H^+^ efflux and reducing extracellular pH, thereby exacerbating TME acidification. Clinical analysis revealed that high expression of NHE1 and CA9 correlated with poor prognosis in PDAC patients. Rimegepant, a CGRP receptor antagonist, inhibited orthotopic tumor growth and exhibited synergistic effects with gemcitabine.

**Conclusion and Discussion:**

In conclusion, our findings uncover a functional connection between CGRPergic sensory neurons and the acidic TME, identifying the CGRP signaling pathway as a potential therapeutic target for PDAC. Despite these findings, the mechanistic conclusions remain correlative and require further validation, and the generalizability to human PDAC warrants future investigation.

## 1 Introduction

Pancreatic ductal adenocarcinoma (PDAC) remains one of the most lethal malignancies worldwide, with a 5-year overall survival rate survival rate of approximately 13% (Verkolf et al., 2025). Despite advances in surgical techniques and adjuvant therapies, the prognosis for PDAC patients has improved marginally over the past decades, mainly due to late diagnosis, early metastasis, and resistance to chemotherapy (Zeng et al., 2019). PDAC is a highly fibrotic and hypovascular solid tumor that creates a hypoxic and acidic tumor microenvironment (TME), which plays a critical role in cancer progression (Thakur et al., 2025). The acidic TME promotes tumor invasion and metastasis, modulates immune cell function, and contributes to therapeutic resistance. Therefore, investigating the underlying molecular mechanisms involved in TME acidification may offer new opportunities for pancreatic cancer therapy.

The peripheral nervous system has emerged as a key component of the tumor microenvironment and contributes to cancer progression (Hanahan and Monje, 2023). Calcitonin gene-related peptide (CGRP) is a neuropeptide predominantly released from sensory nerve endings and has been traditionally studied in the context of vasodilation and migraine pathogenesis (Russo and Hay, 2023). Recent studies have demonstrated that CGRP can promote tumor growth in several cancer types, including gastric cancer (Zhi et al., 2025), breast cancer (Zhang et al., 2026), and PDAC (Wang et al., 2025), by stimulating the extracellular matrix and modulating immune responses. PDAC is characterized by prominent perineural invasion, and the interactions between tumor cells and nerves play a critical role in the development, progression, and pain generation of this disease (Crispim et al., 2026). Notably, the acidic TME has been shown to induce the generation of CGRPergic neurons, suggesting a potential interplay between tumor acidity and neural signaling (Yoneda et al., 2011). However, the functional role of CGRP in PDAC and its involvement in regulating the acidic TME remain largely unexplored.

The maintenance of acidic pH in the TME is primarily orchestrated by a network of proton transporters, including Na^+^/H^+^ exchangers, H^+^/K^+^ ATPases, monocarboxylate transporters, bicarbonate transporters and carbonic anhydrases (Pedersen et al., 2017; Swietach et al., 2023). These proteins facilitate the extrusion of protons from cancer cells, thereby promoting extracellular acidification while maintaining intracellular pH homeostasis. One recent study has demonstrated that inhibition of the bicarbonate transporter SLC4A4 alleviates TME acidosis caused by extracellular bicarbonate accumulation in pancreatic cancer cells, thereby suppressing tumor growth and metastasis (Cappellesso et al., 2022). Whether CGRP regulates the expression or activity of these proton transporters in PDAC remains unknown.

In this study, we investigated the role of CGRP in PDAC progression, with a particular focus on its regulation of the acidic TME. Mechanistically, CGRP establishes an acidic microenvironment by modulating the expression of NHE1 and CA9, thereby promoting PDAC cell proliferation and migration. Moreover, the CGRP receptor antagonist Rimegepant suppresses tumor growth in orthotopic PDAC models and synergizes with gemcitabine. Our findings partially reveal a previously unrecognized correlation between CGRP signaling and tumor acidosis, supporting CGRP as a promising therapeutic target for PDAC.

## 2 Methods

### 2.1 Animal protocol

All animal experiments were approved by the Medical Animal Welfare Committee of the Second Xiangya Hospital (No. 20240773), and were conducted in accordance with institutional guidelines. CGRP knockout (CGRP^KO^/CGRP^−/−^) C57BL/6 mice were generated using CRISPR/Cas9 technology, which genotypes were verified by agarose gel electrophoresis (Supplementary Figure S1). Eight-week-old wild-type (WT) C57BL/6 mice and CGRP^KO^ mice were housed in specific pathogen-free barrier facility with free access to food and water. Mice were randomly assigned to experimental groups. The PANC02 cell line was authenticated by STR profiling and confirmed negative for *mycoplasma* contamination using a *mycoplasma* detection kit. PANC02 cells at logarithmic growth phase were harvested, resuspended in PBS at a density of 5 × 10^6^ cells per 40 μL, and injected into the tail of the pancreas. Tumor growth was monitored by palpation, once tumors had established, mice were randomly assigned. Gemcitabine (Gem) was dissolved in saline and administered intraperitoneally at 20 mg/kg every other day. Rimegepant (Rim, 20 mg/kg) was formulated in a vehicle containing 2% DMSO, 40% PEG300, 5% Tween-80, and 45% saline, and was administered daily by oral gavage. At the endpoint, tumors were excised, weighed, and processed for histological and molecular analyses. Tumor volume measurements, histological scoring, and immunohistochemical evaluations were performed by investigators blinded to the group allocation to ensure objectivity.

### 2.2 Cell counting Kit-8 (CCK8)

The murine pancreatic cancer cell line PANC02 was cultured in Dulbecco’s modified Eagle’s medium supplemented with 10% fetal bovine serum and 1% penicillin/streptomycin at 37 °C with 5% CO_2_. Cells in the logarithmic growth phase were harvested, seeded into 96-well plates at a density of 8 × 10^3^ cells per well, and treated with CGRP (0.01–5 μM) for 48 h. Subsequently, 10% CCK8 solution was added to each well and incubated for 1 h. Absorbance was measured at 450 nm using a microplate reader.

### 2.3 Wound healing assay

PANC02 cells were seeded into 6-well plates and cultured to 90% confluence. A sterile 200 μL pipette tip was used to create a linear scratch. After washing with PBS to remove detached cells, the cells were incubated with serum-free medium containing CGRP (0, 0.01, 0.1, 0.5 μM) for 24 h and 48 h. Wound closure was photographed under an inverted microscope, and the migration area was quantified using ImageJ software.

### 2.4 Cell proliferation assay

Cell proliferation was evaluated using the 5-ethynyl-2′-deoxyuridine (EdU) incorporation assay. PANC02 cells seeded in 24-well plates were treated with CGRP (0, 0.01, 0.1, 0.5 μM) for 48 h, followed by incubation with 10 μM EdU for an additional 2 h. Cells were then fixed, permeabilized, and counterstained with Hoechst 33,342. EdU-positive cells were visualized under a fluorescence microscope, and the percentage of EdU-positive cells was calculated using ImageJ software.

### 2.5 Extracellular pH measurement

Extracellular pH was measured using a microelectrode pH meter. PANC02 cells were seeded in 24-well plates and treated with CGRP (0.5 μM), or CGRP plus Rimegepant (1 μM) for 48 h. The culture medium was collected, and pH was measured immediately under sterile conditions at 37 °C.

### 2.6 Combination index (CI) analysis

Both Rimegepant and gemcitabine were tested individually and in combination at concentrations of 0, 0.001, 0.01, 0.1, 1, and 10 μM. The combination group was prepared at a fixed concentration ratio (1:1) with serial dilutions. After 48 h of treatment, cell viability was determined by CCK-8 assay, and the fraction affected (Fa) at each concentration was calculated. Based on the Chou-Talalay median-effect method, the combination index (CI) was calculated using CompuSyn software. Concentration and corresponding Fa values for single agents and their combination were input into the software. CI < 0.7 was considered strong synergism.

### 2.7 Hematoxylin and eosin (H&E) staining

Fixed in 4% paraformaldehyde and embedded in paraffin, pancreatic tissues were sliced at a thickness of 4 μm. Slices were deparaffinized, rehydrated, and stained with hematoxylin and eosin. Histological evaluation was performed under a light microscope to assess tumor morphology and stromal reaction.

### 2.8 Immunohistochemistry (IHC)

Paraffin-embedded tissue slices were subjected to antigen retrieval and incubated with primary antibodies against Ki67 (1:200), CGRP (1:100) overnight at 4 °C. After incubation with horseradish peroxidase (HRP)-conjugated secondary antibody, signals were visualized using diaminobenzidine and counterstained with hematoxylin. Immunostaining was scored based on staining intensity and the percentage of positive cells. Staining intensity was graded as 0 (negative), 1 (weak), 2 (moderate), or 3 (strong), and the percentage of positive cells was recorded as 1(0%–25%), 2(25%–50%), 3(50%–75%), 4(75%–100%). IHC score = intensity score × percentage of positive cells. All sections were independently scored by two investigators blinded to the group allocation, and the average values were used for statistical analysis.

### 2.9 Bioinformatics analysis

Transcriptomic data from the GSE71729 dataset were downloaded from the Gene Expression Omnibus (GEO) database to analyze the expression of proton transporters in human pancreatic cancer tissues compared with normal pancreatic tissues. Protein expression data of NHE1 and CA9 in pancreatic cancer and normal tissues were obtained from The Human Protein Atlas. Overall survival analysis and correlation analysis between NHE1 and CA9 were performed using the Gene Expression Profiling Interactive Analysis (GEPIA 2) database based on TCGA and GTEx datasets. Gene expression data of PAAD were obtained from the UCSC Xena database. Patients were stratified into CGRP-high and CGRP-low groups based on CGRP expression levels, followed by differential expression analysis and Gene Set Enrichment Analysis (GSEA). Protein-protein interaction (PPI) network was visualized by String and Cytoscape software.

### 2.10 qRT-PCR

Total RNA was extracted from tumor tissues using TRIzol reagent and reverse-transcribed into cDNA using the Evo M-MLV Reverse Transcription Kit (Accurate Biology, AG11705). qRT-PCR was performed using the SYBR Green Pro Taq HS Premix (Accurate Biology, AG11701) on a real-time PCR system. The primer sequences (CA9/NHE1/ATP4A/MCT4/GAPDH) used are listed in Supplementary Figure S1.

### 2.11 Western blotting (WB)

Proteins were extracted from pancreatic tissues using lysis buffer (RIPA buffer: protease inhibitor: phosphatase inhibitor = 100: 1: 1). Protein (20 μg) was separated by SDS-PAGE and transferred onto PVDF membranes. The membranes were blocked with 5% non-fat milk and incubated with primary antibodies against p-ERK, ERK, p-CREB, CREB, NHE1, CA9, and GAPDH overnight at 4 °C. After incubation with HRP-conjugated secondary antibodies, signals were detected using enhanced chemiluminescence and visualized with a chemiluminescence imaging system.

### 2.12 Data analysis

All data are presented as mean ± standard error. Normality was assessed using the Shapiro-Wilk test, and homogeneity of variances was evaluated using the Brown-Forsythe test. For data conforming to normal distribution, intergroup comparisons were performed using one-way analysis of variance (ANOVA) followed by Tukey’s post-hoc test for multiple comparisons. For data not following a normal distribution, the non-parametric Mann-Whitney U test was employed for between-group comparisons. Survival curves were estimated using the Kaplan-Meier method and compared by log-rank test. Correlation analysis was performed using Pearson’s correlation coefficient. All statistical analyses were conducted using GraphPad Prism 9.0, and *P* < 0.05 was considered statistically significant.

## 3 Results

### 3.1 CGRP knockout suppressed orthotopic pancreatic tumor growth in mice

To investigate the role of CGRP in pancreatic cancer progression, we established the PDAC model using CGRP^KO^ mice. CGRP deficiency significantly reduced tumor growth compared with WT mice (Figure 1A). Histological analysis by H&E staining revealed diminished pancreatic ductal/stromal response in CGRP^KO^ mice (Figures 1B,D). IHC staining further demonstrated decreased Ki67 expression in pancreatic tumors from CGRP^KO^ mice, indicating reduced tumor cell proliferation (Figure 1C). Collectively, these findings suggest that CGRP may serve as a critical regulator of PDAC progression.

**FIGURE 1:**
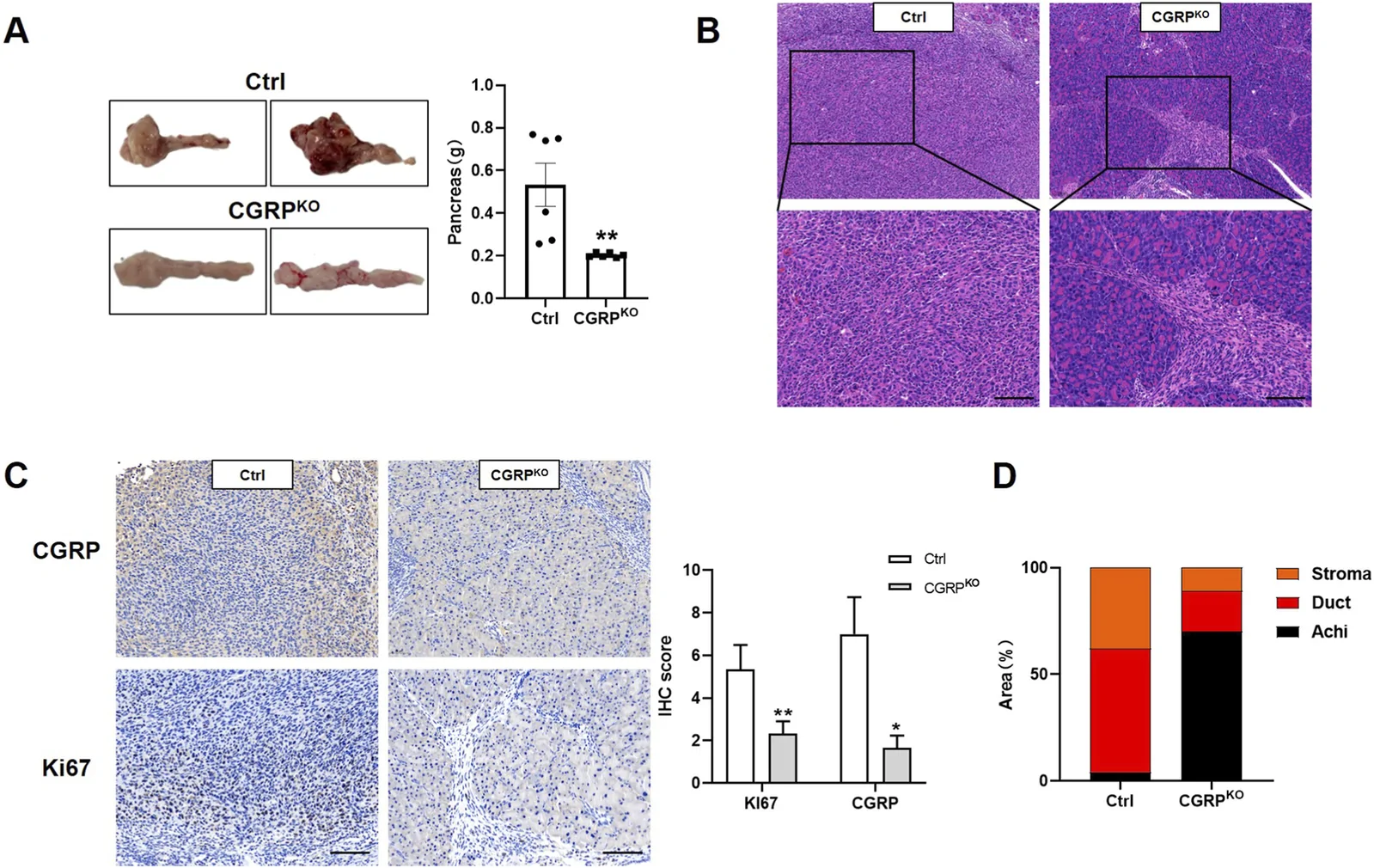
CGRP knockout suppressed orthotopic pancreatic tumor growth in mice. **(A)** Representative photographs of pancreas with tumors and quantitative analysis of pancreas weight; **(B)** Representative pictures of HE staining; **(C)** Representative captures of immunocytochemistry indicated CGRP and Ki67 expression in PDAC tissues from CGRP^KO^ and WT mice, and quantitative analysis of IHC score; **(D)** Histological analysis of PDAC tissues from CGRP^KO^ and WT mice. Scale bar: 100 μm. Data are presented as mean ± SEM (n = 3–6). ^*^ *P* < 0.05, ^**^ *P* < 0.01 vs. Ctrl.

### 3.2 Exogenous CGRP promoted PANC02 cells proliferation

To further elucidate the role of CGRP in PDAC progression, PANC02 cells were treated with exogenous CGRP for 24h or 48 h. CCK8 assay revealed that CGRP at concentrations ranging from 0.01 to 5 μM enhanced cell viability (Figure 2A), with significant effects observed at 0.01, 0.1, and 0.5 μM, which concentrations were therefore selected for subsequent experiments. Wound healing assays demonstrated that CGRP promoted the migratory capacity of PANC02 cells at both 24 and 48 h (Figures 2B,C), which was further confirmed by Transwell migration assays (Figures 2D,F). EdU staining showed that CGRP significantly increased the percentage of EdU-positive cells (Figures 2D,E). In summary, these results indicate that exogenous CGRP enhances the proliferation and migration of PANC02 cells, supporting a pro-tumorigenic role of CGRP in PDAC progression.

**FIGURE 2:**
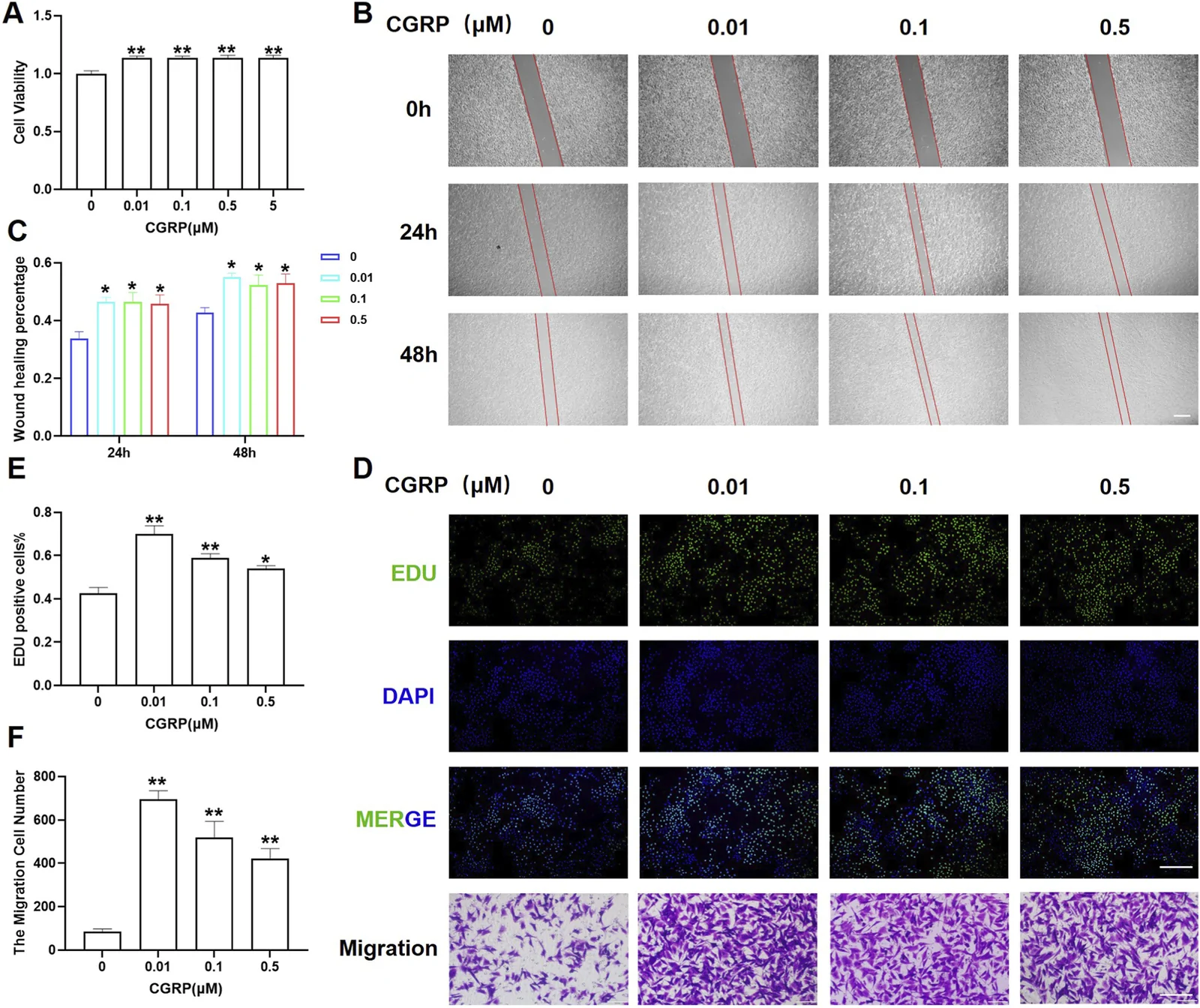
Exogenous CGRP promoted PANC02 cells proliferation. **(A)** Cell viability analysis of PANC02 cells after treatment with CGRP for 48h; **(B,C)** Representative captures of wound healing and quantitative analysis of wound healing percentage; **(D)** Representative pictures of EdU staining and Transwell migration; **(E)** Quantitative analysis of EdU-positive cells percentage; **(F)** Quantitative analysis of the migration cell number. Scale bar: 100 μm. Data are presented as mean ± SEM (n = 3). ^*^ *P* < 0.05, ^**^ *P* < 0.01 vs. CGRP (0 μM). ns, not significant.

### 3.3 CGRP lowered extracellular pH by upregulating NHE1 and CA9 in pancreatic cancer cells

Previous studies have demonstrated that the acidic tumor microenvironment promotes the generation of CGRPergic neurons (Yoneda et al., 2011; Hiasa et al., 2017). IHC analysis revealed elevated expression of both Ki67 and CGRP in pancreatic cancer tissues (Figure 3A). Consistently, Western blotting showed increased phosphorylation of ERK and CREB, indicating enhanced CGRPergic neuron activity in pancreatic cancer (Figure 3B). To identify downstream pathways associated with CGRP signaling, we analyzed the TCGA-PAAD dataset via the UCSC Xena platform. Patients were stratified into CGRP-high and CGRP-low groups based on the upper and lower quartiles of CGRP expression, respectively. Differential expression analysis followed by GSEA revealed significant enrichment of two gene ontology terms: “GOBP_REGULATION_OF_PH” and “GOBP_REGULATION_OF_CELLULAR_PH” (Figures 3C–E). Furthermore, PPI network analysis identified SLC9A1 (NHE1), CA9, and CA2 as core hub proteins, all of which are known to be involved in proton/pH regulation (Figure 3F). To assess whether CGRP contributes to the acidic microenvironment, we analyzed proton transporter expression using the GSE71729 transcriptomic dataset (Figure 3G). Compared with normal samples, pancreatic cancer tissues exhibited marked upregulation of NHE1, CA9, and MCT4, whereas ATP4A was significantly downregulated (Figure 3H). We further examined their expression in CGRP^KO^ and WT mice by qRT-PCR. CGRP deficiency led to reduced mRNA levels of NHE1 and CA9 (Figure 3I), suggesting that CGRP may regulate the acidic microenvironment through transcriptional control of these transporters. To directly test whether CGRP influences extracellular pH, we treated Panco2 cells with exogenous CGRP alone or in combination with Rimegepant. Exogenous CGRP significantly decreased extracellular pH, whereas co-administration of Rimegepant reversed this effect (Figure 3J). These findings demonstrate that CGRP lowers extracellular pH via upregulation of NHE1 and CA9, thereby promoting an acidic TME. This acidic milieu may in turn facilitate the generation of CGRPergic neurons, possibly forming a correlation that sustains tumor progression.

**FIGURE 3:**
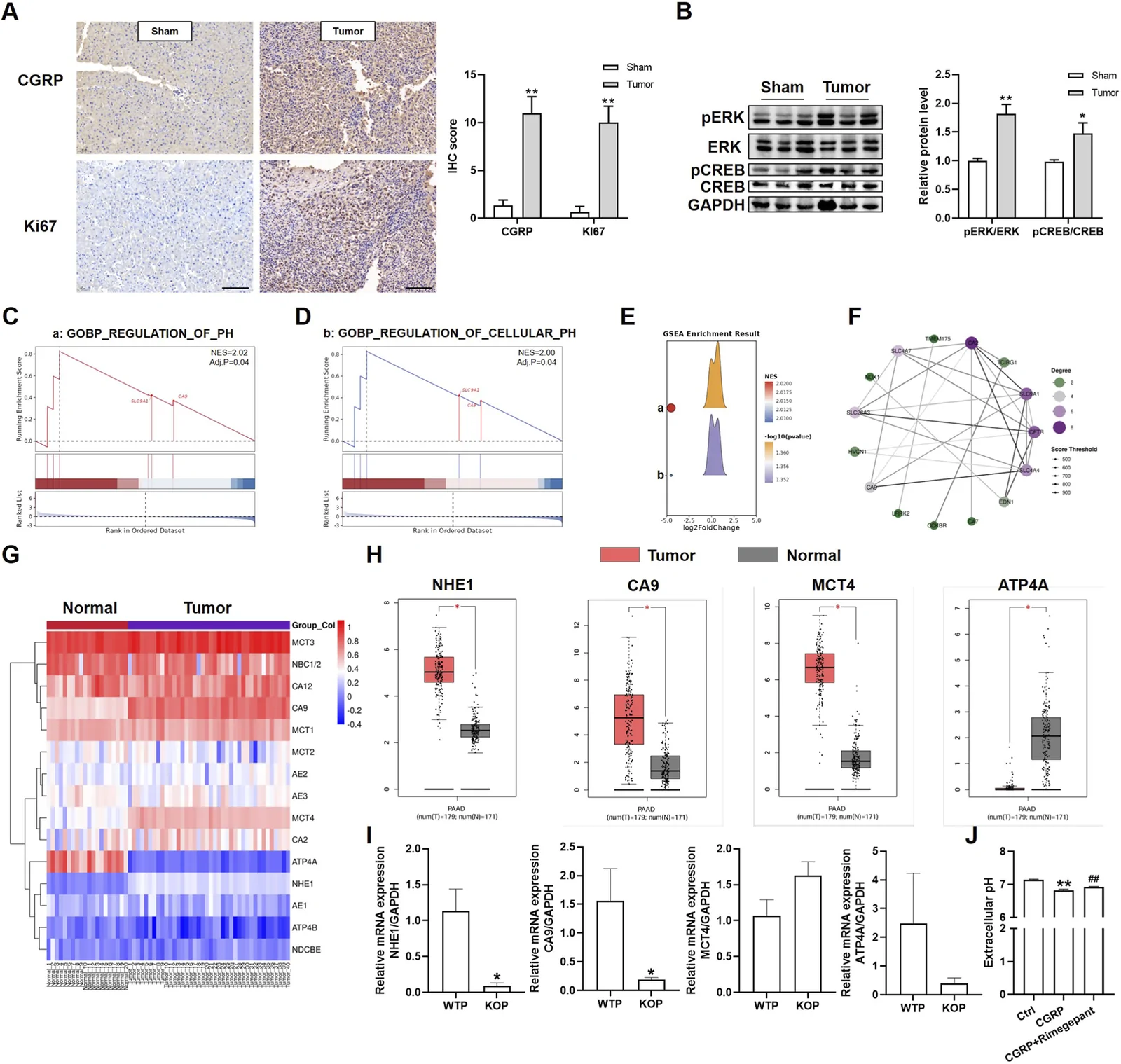
CGRP lowered extracellular pH by upregulating NHE1 and CA9 in pancreatic cancer cells. **(A)** Representative captures of immunocytochemistry indicated CGRP and Ki67 expression in pancreatic tissues, and quantitative analysis of IHC score; **(B)** WB analysis of p-ERK and p-CREB protein expression in PDAC tissues; **(C)** Patients were stratified into CGRP-high (upper quartile) and CGRP-low (lower quartile) groups based on CGRP expression. GSEA revealed significant enrichment of “GOBP_REGULATION_OF_PH” terms in the CGRP-high vs. CGRP-low group. **(D)** GSEA revealed significant enrichment of “GOBP_REGULATION_OF_CELLULAR_PH” terms in the CGRP-high vs. CGRP-low group. **(E)** GSEA enrichment results in the CGRP-high vs. CGRP-low group. **(F)** PPI network analysis further identified SLC9A1 (NHE1), CA9, and CA2 as core hub proteins within the proton/pH regulatory network; **(G)** Heatmap of proton transporters expression within the tumor from PDAC patients compared with normal controls using TCGA datasets; **(H)** The expression of NHE1, CA9, MCT4 and ATP4A within the tumor from PDAC patients compared with normal controls using TCGA datasets; **(I)** The mRNA level of NHE1, CA9, MCT4 and ATP4A in PDAC tissues from CGRP^KO^ and WT mice were analyzed by qRT-PCR; **(J)** Extracellular pH in PANC02 cells after treatment with CGRP (0.5 μM) and Rimegepant (1 μM) for 48 h. Scale bar: 100 μm. Data are presented as mean ± SEM (n = 3–6). ^*^ *P* < 0.05, ^**^ *P* < 0.01 vs. Ctrl.

### 3.4 Upregulation of NHE1 and CA9 correlated with poor prognosis in human PDAC

Our preclinical findings indicated that CGRP-mediated regulation of CA9 and NHE1 promotes the proliferation and migration of PANC02 cells. To investigate the clinical significance of CA9 and NHE1 in PDAC, we performed bioinformatics analysis using The Human Protein Atlas and GEPIA 2 databases. IHC staining from The Human Protein Atlas showed higher expression levels of CA9 and NHE1 in PDAC tissues compared with normal pancreatic tissues (Figures 4A,C). Overall survival analysis revealed that high expression of either CA9 or NHE1 was associated with shorter survival in PDAC patients (Figures 4B,D). Furthermore, correlation analysis demonstrated a positive correlation between CA9 and NHE1 expression (Figure 4E). Overall, these analyses suggest that CA9 and NHE1 may serve as potential oncogenic factors in human PDAC. CGRP exerts its biological effects through binding to a heterodimeric receptor complex composed of calcitonin receptor-like receptor (CLR) and receptor activity-modifying protein 1 (RAMP1) (Zhi et al., 2025). To confirm the functional relevance of this receptor complex in pancreatic cancer cells, we first examined the expression of RAMP1, RAMP2, and RAMP3 in Panc02 cells, and found that RAMP1 was the predominant isoform expressed (Figure 4G). Analysis of the TCGA-PAAD dataset further revealed that CLR was significantly upregulated in PDAC tissues compared with normal controls, and its expression positively correlated with both NHE1 and CA9 (Figures 4F,H,I). Collectively, these findings support that CGRP signaling in PDAC is mediated through CLR/RAMP1.

**FIGURE 4:**
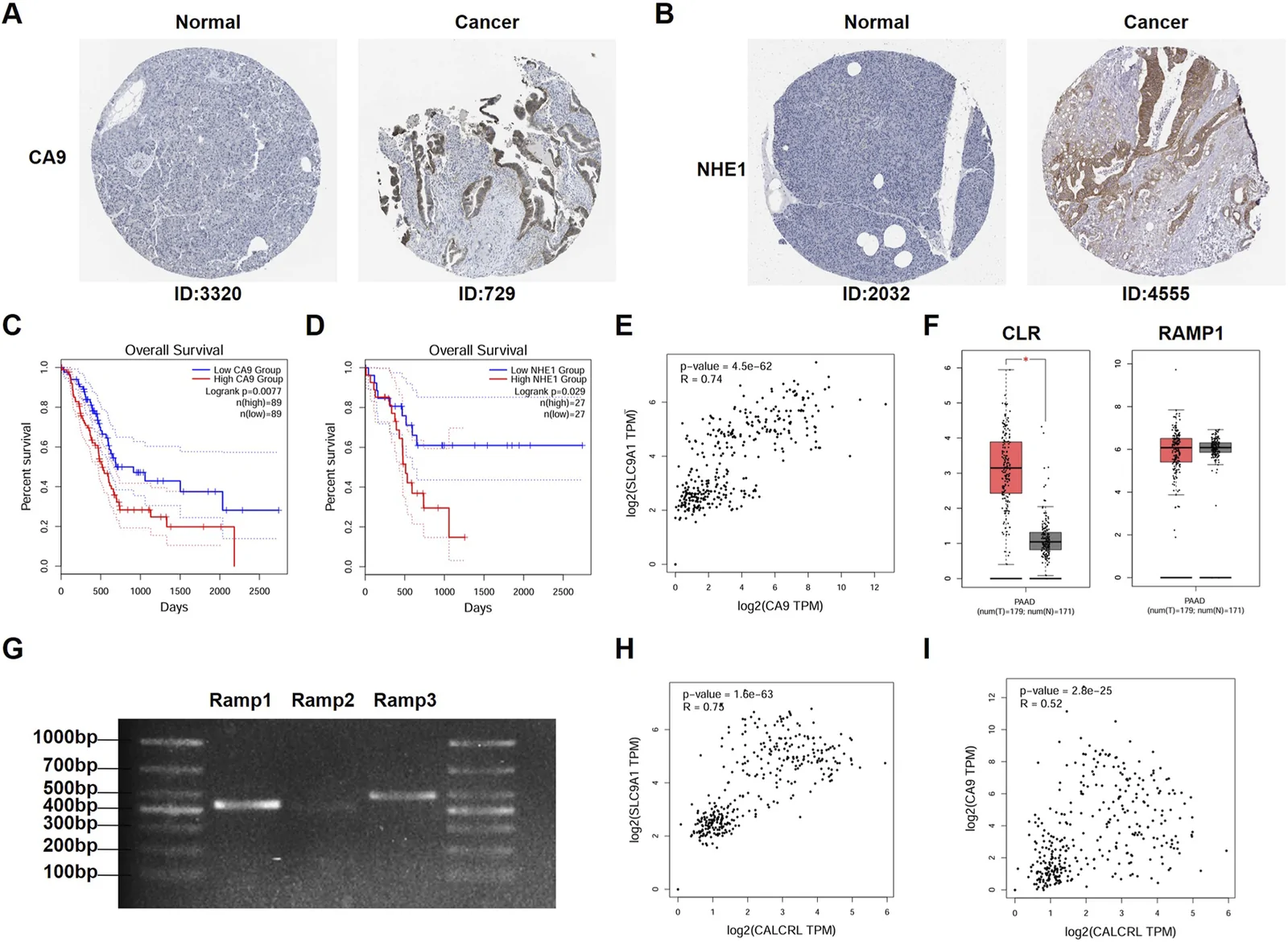
Upregulation of NHE1 and CA9 correlated with poor prognosis in human PDAC. **(A)** The upregulation of CA9 protein expression within the tumor from PDAC patients compared with normal controls using the Human Protein Atlas database; **(B)** The upregulation of NHE1 protein expression within the tumor from PDAC patients compared with normal controls using the Human Protein Atlas database; **(C)** Kaplan-Meier survival analysis of CA9 gene expression in PDAC patients using TCGA datasets; **(D)** Kaplan-Meier survival analysis of NHE1 gene expression in PDAC patients using TCGA datasets; **(E)** The correlation of NHE1 and CA9 in PDAC patients using TCGA datasets; **(F)** The expression of CLR and RAMP1 within the tumor from PDAC patients compared with normal controls using TCGA datasets; **(G)** The correlation of CLR and NHE1 in PDAC patients using TCGA datasets; **(H)** The correlation of CLR and CA9 in PDAC patients using TCGA datasets; **(I)** The expression of Ramp1, Ramp2 and Ramp3 in PANC02 detected by agarose gel electrophoresis.

### 3.5 Rimegepant synergized with gemcitabine to inhibit pancreatic cancer growth in mice

To investigate the potential synergistic antitumor effects of Rimegepant, the CGRP receptor antagonist, in combination with gemcitabine, we established orthotopic PDAC mouse models using both CGRP^KO^ and WT mice. The mice were treated with Rimegepant, gemcitabine, or their combination (Figure 5A). Compared with WT controls, both Rimegepant and gemcitabine monotherapy significantly reduced tumor growth, and the combination therapy exhibited superior antitumor efficacy (Figures 5B,C). IHC staining further revealed decreased Ki67 expression in pancreatic tumors following treatment with Rimegepant, gemcitabine, or their combination, indicating reduced tumor cell proliferation (Figure 5D). Notably, the antitumor efficacy of Rimegepant was comparable to that of CGRP knockout. WB analysis showed that both CGRP knockout and Rimegepant treatment suppressed the expression of CA9 and NHE1 (Figure 5E). The above findings conclude that CGRP knockout inhibits PDAC progression through downregulation of CA9 and NHE1. As shown in Figure 5F, the combination of Rimegepant and gemcitabine achieved an inhibition rate of 70%, which was markedly higher than that of Rimegepant alone (40%) or gemcitabine alone (50%). Moreover, to quantitatively evaluate the combined effect of Rimegepant and gemcitabine, we performed CI analysis using CompuSyn software based on the Chou-Talalay method in PANC02 cells. Cells were treated with Rimegepant and gemcitabine alone or in combination at a fixed concentration ratio across a range of doses. The median-effect plot showed a linear relationship between the logarithm of the dose and the logarithm of the fraction affected (Fa) for both single agents and their combination (r > 0.95), indicating that the experimental data were well-fitted to the Chou-Talalay model (Figure 5G). The dose-effect curves demonstrated that both drugs exhibited a concentration-dependent inhibitory effect on PANC02 cell viability, and the combination treatment resulted in a leftward shift of the dose-effect curve compared with either monotherapy (Figure 5H). The CI values at various effect levels are shown in the Fa-CI plot (Figure 5I). Notably, the CI values were consistently below 0.2 across all tested effect levels, indicating a strong synergistic effect between Rimegepant and Gemcitabine in PANC02 cells.

**FIGURE 5:**
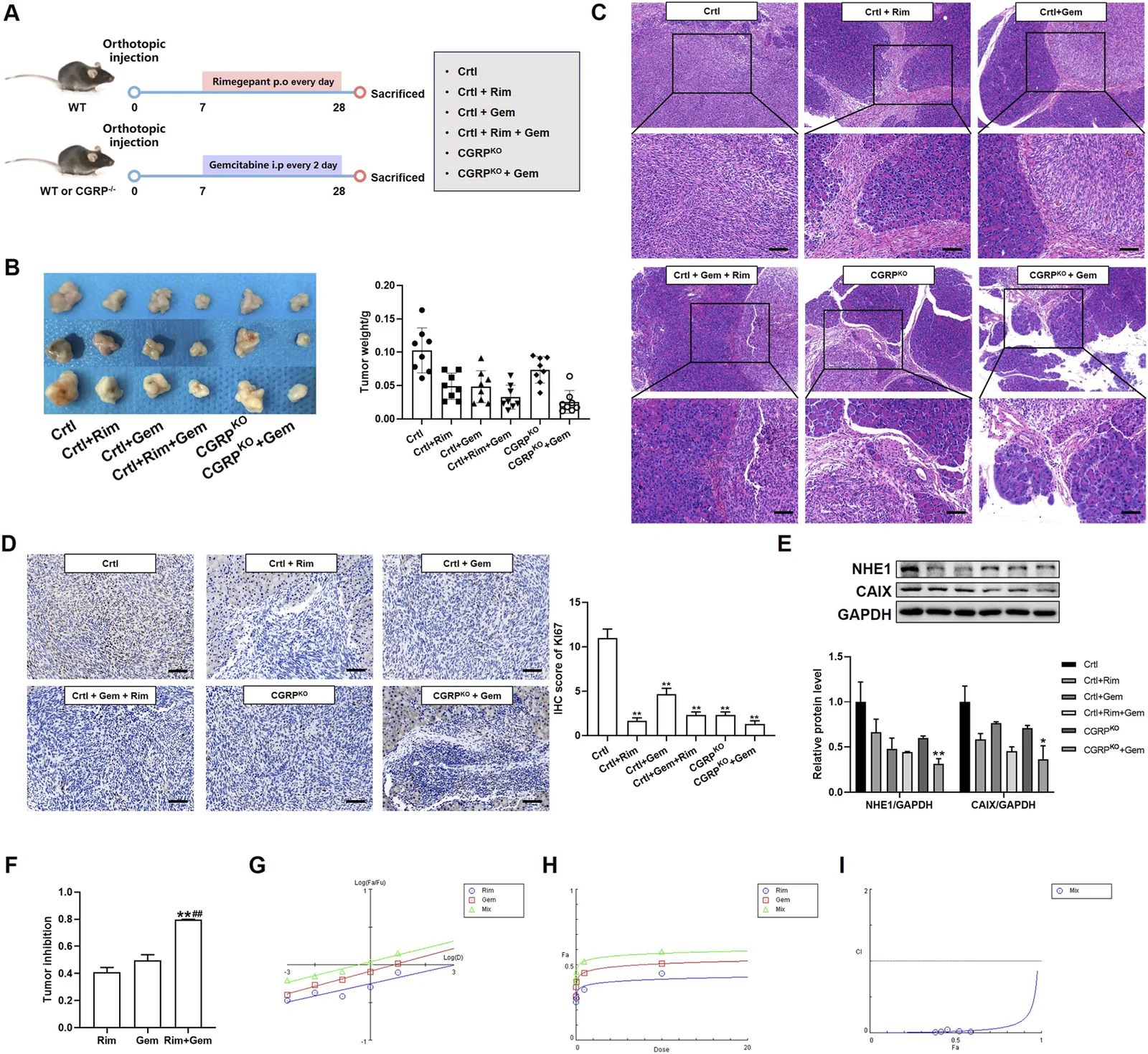
Rimegepant synergized with Gemcitabine to inhibit pancreatic cancer growth in mice. **(A)** Schematic of treatment regimen and group allocation; **(B)** Representative photographs of isolated tumors and quantitative analysis of tumor weight; **(C)** Representative pictures of HE staining; **(D)** Representative captures of immunocytochemistry indicated Ki67 expression in PDAC tissues and quantitative analysis of IHC score; **(E)** WB analysis of CA9 and NHE1 protein expression in PDAC tissues. **(F)** Tumor growth inhibition rates in an orthotopic PDAC mouse model; **(G)** Median-effect plots showing the linear relationship between log (dose) and log (Fa) for Rimegepant, gemcitabine, and their combination. Cells were treated with indicated concentrations for 48 h, and cell viability was measured by CCK-8 assay; **(H)** Dose-effect curves of Rimegepant, gemcitabine, and their combination at a fixed ratio; **(I)** Fa-CI plot showing combination index (CI) values at different effect levels. CI < 1 indicates synergism, CI < 0.7 indicates strong synergism. Scale bar: 100 μm. Data are presented as mean ± SEM (n = 3–6). ^*^ *P* < 0.05, ^**^ *P* < 0.01 vs. Ctrl; ^**^ *P* < 0.01 vs. Rim, ^##^ *P* < 0.01 vs. Gem.

## 4 Discussion

This study provides the first evidence suggesting that CGRP promotes pancreatic cancer progression by regulating NHE1 and CA9. Nevertheless, several limitations should be acknowledged. Firstly, although we demonstrated downregulation of NHE1 and CA9 protein expression following CGRP knockout, and GSEA and PPI analyses provided correlative evidence in PDAC, rescue experiments using specific inhibitors (e.g., the NHE1 inhibitor Cariporide (Liu et al., 2026) or the CA9 inhibitor Acetazolamide (Chen et al., 2023) were not performed to further strengthen the causal relationship between CGRP and the two transporters. Moreover, this study relies primarily on the PANC02 cell line and whole-body CGRP knockout mice, without comprehensive validation across multiple human PDAC cell lines and primary human tumor samples, which limits the generalizability and translational relevance of our findings. Additionally, although the positive feedback loop hypothesis is supported by our findings and existing literature, direct experimental evidence is still lacking. Future studies combining *in vitro* co-culture systems, conditional gene knockout animal models, and *in vivo* imaging techniques are warranted to further validate this hypothesis.

The interplay between the nervous system and tumors has emerged as a frontier in TME research, positioning therapeutic strategies that target this crosstalk as a promising new cornerstone of cancer treatment. The pancreas was densely innervated by both sympathetic and parasympathetic nerves, and the development of pancreatic cancer involved a close malignant interaction with the nervous system, characterized by prominent perineural invasion and pain (Mancusi and Monje, 2023). As a key neuropeptide released from sensory nerve endings, CGRP has been shown to exert pro-tumorigenic effects in various malignancies. For instance, neurons promoted gastric tumor growth and metastasis by secreting CGRP, whereas gastric tumor cells reciprocally activated neurons to release more CGRP, which was a vicious cycle that positioned nociceptive neurons or the CGRP signaling pathway as potential therapeutic targets in gastric cancer (Zhi et al., 2025). In the present study, we demonstrated that exogenous CGRP significantly promoted the proliferation and migration of PANC02 cells *in vitro*, whereas CGRP knockout suppressed pancreatic tumor progression. However, a recent study reported that CGRP did not directly affect the apoptosis or proliferation of KPC cells, but instead interacted with cancer-associated fibroblasts to inhibit IL-15 expression and impaired the anti-tumor function of natural killer cells, thereby facilitating tumor progression and cancer pain (Wang et al., 2025). This discrepancy may be attributed to differences in cell lines and the species specificity of CGRP. Notably, we provided strong evidence demonstrating that CGRP promotes proliferation and migration in pancreatic cancer, as evidenced by both exogenous CGRP supplementation *in vitro* and CGRP knockout *in vivo*.

In addition, our study further revealed significantly elevated CGRP expression in pancreatic cancer tissues, accompanied by increased levels of the neuronal activation markers p-ERK and p-CREB, indicating that CGRPergic neurons were activated in pancreatic cancer. This observation was consistent with previous reports demonstrating that an acidic TCM could induce the generation of CGRPergic neurons (Yoneda et al., 2011). Mechanistically, we found that CGRP upregulated the expression of the proton transporters NHE1 and CA9, probably facilitating H^+^ efflux from tumor cells and thereby exacerbating extracellular acidification (as shown in Figure 6). Based on these findings, we propose a possible positive feedback loop hypothesis: the acidic TME induced the activation of CGRPergic neurons; the released CGRP directly promoted tumor cell proliferation, and further reduced extracellular pH by upregulating NHE1 and CA9, thereby intensifying microenvironmental acidity, which in turn promoted the generation of CGRPergic neurons, forming a vicious cycle that driven pancreatic cancer progression. However, measurement of extracellular pH alone is insufficient to fully validate this positive feedback loop, and the underlying mechanisms warrant further investigation.

**FIGURE 6:**
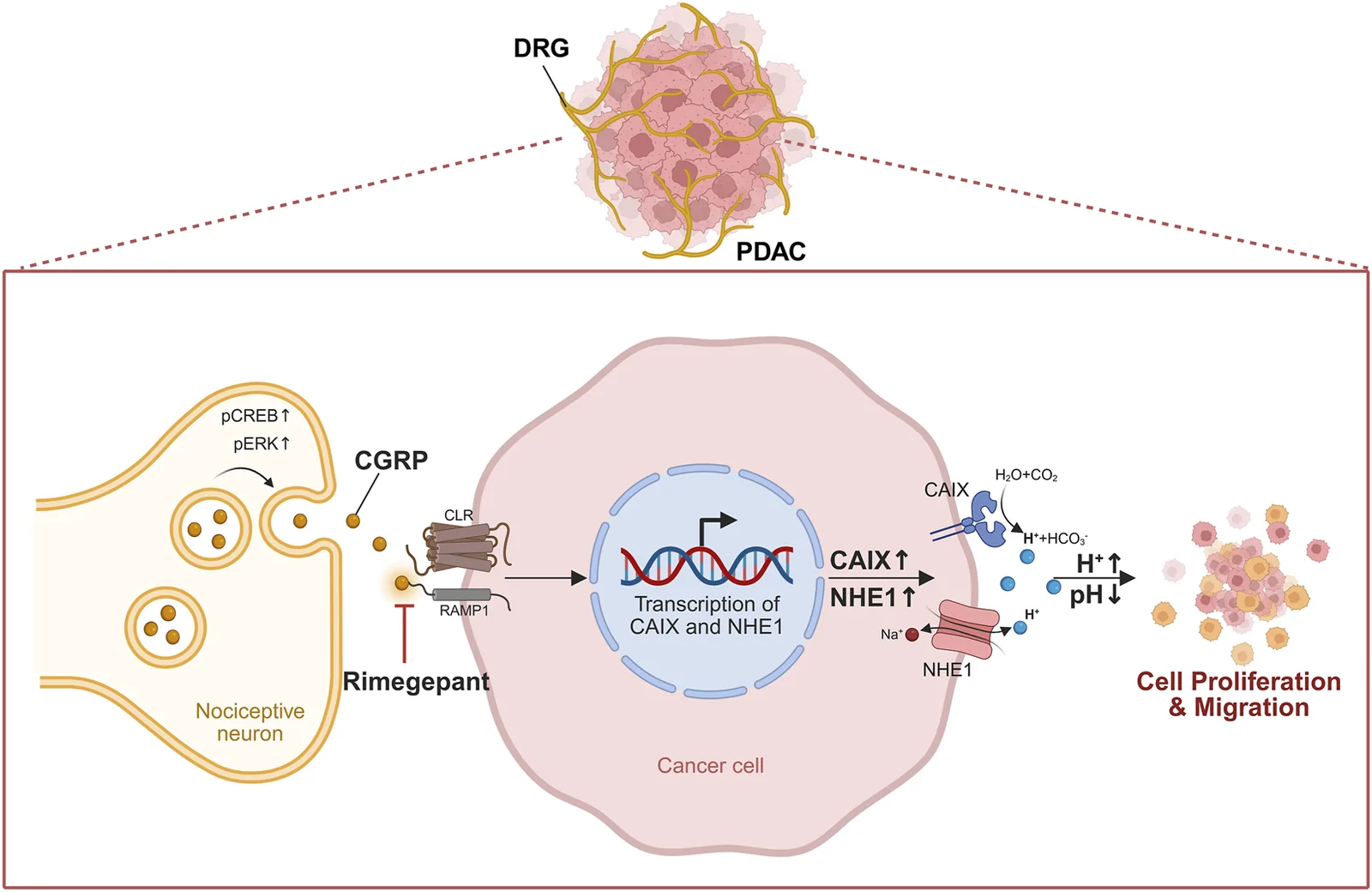
CGRP upregulates NHE1 and CA9 to induce an acidic tumor microenvironment, promoting PDAC progression. Pharmacological inhibition of CGRP signaling using the receptor antagonist Rimegepant suppresses tumor growth in orthotopic PDAC models, supporting CGRP as a potential therapeutic target in pancreatic cancer.

Based on the finding that CGRP participated in microenvironmental acidification through NHE1 and CA9, we further explored the functional significance of these two proton transporters. NHE1 and CA9 were critical proton transporters that maintain intracellular pH homeostasis and mediate extracellular acidification in tumor cells (Liao et al., 2024). NHE1 extrudes protons via Na^+^/H^+^ exchange, whereas CA9 catalyzes the hydration of CO_2_ to generate H^+^ and HCO_3_^−^, cooperatively contributing to tumor microenvironment acidification (Parks et al., 2017). However, functional activity of NHE1 and CA9 (e.g., H^+^ flux rate, enzymatic activity) has not been directly assessed in our study. Numerous studies have reported that these two transporters are overexpressed in a variety of malignant tumors, and that suppression of their expression reduces tumor invasion, metastasis, and chemoresistance (Alameri et al., 2025; Hyun et al., 2025; Paoletti et al., 2026; Roshdy et al., 2026). Through bioinformatics analysis and studies using CGRP knockout models, we demonstrated that CGRP positively regulated the transcriptional levels of NHE1 and CA9, and the expression of which was significantly correlated with poor prognosis in patients with pancreatic cancer. However, the precise molecular mechanism by which CGRP regulates NHE1 and CA9 remains unclear. Whether CGRP directly modulates the transcription of these genes through its canonical receptor complex RAMP1-CLR to activate downstream signaling pathways, such as the cAMP-PKA, MAPK/ERK, or CaMK-CREB axes (Sun et al., 2020; Tian et al., 2022; Hamiti et al., 2025), or indirectly influences their expression via other mechanisms, warrants further investigation.

It should be noted that our study relies primarily on the PANC02 cell line and whole-body CGRP knockout mice, and weather our findings can be generalized to human PDAC requires further validation. To preliminarily assess generalizability, we performed proliferation assays in BxPC3 and PANC1 human pancreatic cancer cell lines (Supplementary Figure S2), which confirmed the pro-proliferative effect of CGRP, suggesting that this phenomenon may not be limited to murine cells. Nevertheless, comprehensive validation in a broader panel of human cell lines and primary samples is warranted. Additionally, validation in a larger number size would strengthen the robustness and generalizability of our conclusions.

Regarding the strategies for CGRP signaling intervention, we employed two complementary approaches. While whole-body CGRP knockout effectively eliminates global CGRP effects, it may introduce systemic confounding and cannot fully distinguish tumor-autonomous effects from systemic contributions. This distinction could be addressed in future studies using cell-specific or conditional knockout models. Notably, the CGRP receptor antagonist Rimegepant, a clinically approved oral drug for migraine treatment, exhibits high selectivity for the human CGRP receptor (Ki = 0.027 nM, IC50 = 0.14 nM) (Blair, 2023). In the present study, the pharmacological effects of Rimegepant, including suppression of tumor growth and downregulation of NHE1/CA9 expression, were highly consistent with those observed in CGRP knockout mice. This concordance between genetic and pharmacological approaches strongly suggests that the observed effects are specifically attributable to blockade of CGRP signaling rather than non-specific off-target effects, and provides preclinical evidence supporting the potential repurposing of Rimegepant for PDAC therapy.

## 5 Conclusion

CGRP is upregulated in pancreatic cancer and promotes tumor cell proliferation and migration. Mechanistically, CGRP promotes an acidic tumor microenvironment by upregulating the proton transporters NHE1 and CA9, thereby lowering extracellular pH and promoting PDAC progression. Elevated expression of NHE1 and CA9 correlates with poor prognosis in PDAC patients, highlighting their clinical relevance. Genetic ablation of CGRP or pharmacological inhibition using the CGRP receptor antagonist Rimegepant effectively suppresses tumor growth in orthotopic PDAC models and synergizes with gemcitabine. In summary, these findings suggest a correlation between CGRP signaling and tumor acidosis, and support CGRP as a potential therapeutic target for pancreatic cancer.

## Data Availability

The original contributions presented in the study are included in the article/Supplementary Material, further inquiries can be directed to the corresponding authors.
